# Modern Sociology

**Published:** 1901-12-28

**Authors:** 


### Modern Sociology.

### THE STUDY OF THE CHILD.

While the opposing cries of over-pressure and under-education ring persistently in their ears, those who have to do with the teaching of the young may sometimes be at a loss to judge if they are proceeding on the right lines in their efforts. It is undeniable that some children seem to be overworked at school, and that those very children profit less by the teaching they receive than others to whom their studies offer no special difficulty. It is obvious that this difference of effort and result arises from the difference of capacity in different pupils, but as yet no effort has been made in this country to test systematically the powers of children before assigning them their place in the school and deciding on the work that should be demanded from them. They are placed at first according to their ages, and sometimes even moved on from class to class in accordance with their advance in years rather than their advance in knowledge. The Board of Education of the City of Chicago, on the other hand, has instituted a special department of child-study, which attempts to investigate the mental and physical capacity of each child, with a view to fitting his education to his powers.

Any proper system of child-study must begin with establishing the norm of the average child, in order to fix what children rise above and what fall below the normal. To obtain this, the department we speak of tested the powers of the pupils in three of the largest schools under the Board of Education, choosing schools where the parents of the pupils were in comfortable circumstances, and the majority of them of American birth—altogether the most favourable specimens of childhood to be found in the public schools. The children tested numbered 6,259—2,788 boys and 3,471 girls. The director of the Child-Study Department, Mr. Fred. W. Smedley, expressly protests against the popular notion that the average is the ideal type. Some of the children were as much above the average as others were below; the norm lies between these two extremes. The special points tested were height, both standing and sitting—for the one often differs much from the other—weight, strength as tested by the dynamometer, the vital capacity, which in this connection is used to signify the amount of air that a person can expire after a forced inspiration, and endurance, tested by a machine called an ergograph. This apparatus consists of two parts, a fixing board and a carriage with a tracing apparatus mounted on a suitable frame. The arm is fastened firmly to the fixing board, allowing free movement to only the middle finger of the right hand. To this finger a cord is fastened which, passing over the carriage and over a pulley at the end of the stand, is attached a weight. In each case this weight was 7 per cent. of the weight of the individual. In flexing the finger the weight is lifted, but returns to its original place when the finger is stretched out again. A pen attached to the carriage traces the movement thus made upon a revolving cylinder covered with paper, and a moving tape-line measures the distance the weight has been lifted. The flexion and extension of the hand is timed by a metronome, so that the weight will be lifted 45 times in a minute and a half, which is the time the experiment lasts. It was found that at some point between 90 and 150 seconds a stage of exhaustion was reached at which no child could continue to lift the weight, and 90 seconds was finally decided on as the length of time to be allowed for the test, because it was found that by that time even the strongest pupils would show some signs of fatigue, while only the weakest would be altogether exhausted.

These tests, applied to boys and girls, show such a marked difference between the powers of the two sexes as to make one pause in demanding for girls equal opportunities of education with their brothers. In height, girls keep more nearly level with boys in school years than in most other physical qualities. At the age of two they are a very little shorter than boys, but keep very close upon them till the age of eleven, when they even get ahead, and keep their superiority till about fifteen. Then, however, they begin to lose ground, and while the boy shoots up rapidly and gains several inches between fifteen and twenty, the girl as a rule gains only an inch or two more and stops short in her growth at about seventeen. In the sitting height there is not so much difference as in that taken standing, but in both the boys show a considerable superiority. In weight the same rule holds good. Girls are a little lighter than boys from five to twelve, at twelve they are equal, and from that age to fourteen the girls have the best of it, but after fourteen they gain little, while the boys develop rapidly. It is curious to note, however, that in the matter of strength of hand girls are weaker all along. Even at their best, from five to twelve, a girl's right hand is weaker than a boy's left and after that the difference increases enormously. In fact the girls are nowhere. Of course, one would like to know if a course of study which included golf, tennis, and hockey would not remedy this state of matters a little. The American girl does not go in for athletics. In vital capacity and endurance she shows herself equally inferior to the boys, the difference, as before, becoming more marked as each approaches maturity.

The tests hitherto spoken of are merely physical, and might seem to have nothing to do with intellectual capacity, but taking the average, a healthy well-developed child is more intelligent than a sickly one. A curious fact bearing on this came out in Chicago when the pupils of the "John Worthy" school for children of the criminal classes came to be tested. These boys at the age of nine or ten seemed stronger, heavier, and cleverer than their fellows who had grown up in better conditions, but they lost their superiority then and never regained it. Indeed, the older they grew the more marked became their inferiority to even those of the normal children who were rather below the average in physical and mental attainment. In one point, however, they showed themselves superior. The "John Worthy" boys were more nearly ambidextrous than the normal children. This might be taken to be the result of training in the school of some Chicago Fagin, if it were not that to some extent this ambidexterity seems to go hand in hand with dulness in children of a more fortunate class. In the brightest pupils in all schools the difference of power between the right and left hands seems most marked. This would appear to imply that specialisation is a necessary concomitant of the highest development. In the matter of abnormalities of growth and motor defects, it was found that the children who were dull in class were often those in whom such peculiarities were most marked. Among the dull children there are, of course, to be found those who suffer from defects of sight or hearing, but as the connection between the defect and the failure to profit by instruction is so obvious in these cases, it does not seem worth while to dwell on it. It is clear that a child who does not see or hear well cannot profit by what he is shown or told to the same extent as his normal companion; but the connection between general physical weakness and mental dulness is more suggestive and subtle, even if the conclusions drawn from these Chicago tests cannot be accepted without reserve.

